# Mechanical Outcomes, Microleakage, and Marginal Accuracy at the Implant-Abutment Interface of Original versus Nonoriginal Implant Abutments: A Systematic Review of In Vitro Studies

**DOI:** 10.1155/2018/2958982

**Published:** 2018-12-30

**Authors:** Marco Tallarico, Joseph Fiorellini, Yasushi Nakajima, Yuki Omori, Iida Takahisa, Luigi Canullo

**Affiliations:** ^1^Private Practice, Rome, Italy; ^2^Department of Periodontology, University of Pennsylvania, Pennsylvania, USA; ^3^ARDEC Academy, Ariminum Odontologica, Rimini, Italy; ^4^Department of Oral Implantology, Osaka Dental University, Osaka, Japan

## Abstract

**Purpose:**

Instead of original abutments, compatible abutments are often selected for financial reasons. The present study aimed to evaluate mechanical outcomes, microleakage, and marginal accuracy at the implant-abutment interface of original versus nonoriginal implant abutments.

**Study Selection:**

Search strategy encompassed literature from 1967 up to March 2017 to identify relevant studies meeting the inclusion criteria. The following electronic databases were consulted: PubMed database of the U.S. National Library of Medicine, Embase (Excerpta Medica dataBASE), and the Grey Literature Database (New York Academy of Medicine Grey Literature Report). Quality assessment of the full-text articles selected was performed. Abutments were classified in original (produced by the same implant manufacturer), nonoriginal certified (produced by a third-party milling center, certified by implant companies), and nonoriginal compatible (produced by a third-party milling center for similar connections).

**Results:**

A total of 16 articles fulfilled inclusion criteria and quality assessment and were selected for the qualitative analysis. All of the included studies were in vitro research with high or moderate risk of bias and reported data from 653 implant abutments. Original and nonoriginal certified abutments showed better results in terms of mechanical outcomes, microleakage, and marginal accuracy compared to nonoriginal compatible abutments.

**Conclusions:**

Following the clear warnings coming from the present systematic review, clinical suggestions regarding the effect of a nonoriginal abutment can be drawn. However, in vivo, long-term, randomized controlled trials are needed to provide definitive clinical conclusion about the long-term clinical outcomes of original and nonoriginal abutments.

## 1. Introduction

Current commercially available dental implants are mostly two-part systems: the implant is positioned at the bone level and, once osseointegrated, loaded using an abutment supporting the prosthetic restoration. Microgaps at the implant-abutment interface (IAI) are unavoidable and are the consequences of the micro-tolerances between components required during the industrial manufacturing.

The implant connection has not been demonstrated to prevent bacterial contamination [1, 2]. In contrast, the different torques applied to the abutment-implant system condition the bacterial leakage at the implant interface, with no microleakage observed at 20 and 30 N compared to <10 and 10 N [3]. In reality, the microgap acts as a bacterial reservoir that may trigger an inflammatory host response in the peri-implant soft tissues and bone [1, 2, 4]. More importantly, the gap between the implant and the abutment is a factor in chronic inflammatory infiltration, as it allows the passage of acids, enzymes, bacteria, and/or their metabolic products [5]. In fact, since the IAI is located near the alveolar bone crest, bacterial colonization of the gap has been implicated in the physiological biologic width establishment that occurs during the first 6 months after loading [3, 6]. Interestingly, the 8th consensus of the European Federation of Periodontology [7], as well as the Position Paper of the American Academy of Periodontology [8], agreed upon the likely initial bone remodeling after implant restoration to accommodate biologic width.

The majority of the marginal bone loss (MBL) associated with the biologic width reestablishment has been reported during the first year after implant placement, while hereafter, in patients with adequate levels of oral hygiene, the marginal bone levels stabilize over years. However, micromovements of the implant-abutment complex (directly correlated to the tridimensional micro-tolerances between implant and abutment connection) represent the second key factor for the determination of MBL and biologic width [9]. A recent systematic review of randomized controlled trails [10] and meta-analysis revealed contamination of the IAI, in all patients who received two-piece implant systems. Meta-analysis indicated significant differences in total bacterial count between implants affected by peri-implantitis versus healthy peri-implant tissues, with less bacterial counts in the IAI of healthy patients, for all the investigated gram-negative bacteria except for T. forsythia [10].

In daily practice, practitioners and dental technicians often select compatible abutments for financial reasons. The differences in design are possibly related to patent issues that do not allow for exact replication of components and/or related to the precision level and the quality control of materials used during the manufacturing process. For the latter, nonoriginal components may differ in the design of connecting surfaces, shape, dimensions, and material and have shown higher leakage values [11, 12]. The use of nonoriginal abutments may also increase micromovements at the IAI, and the inner part of the implant may increase the stress on marginal bone level, also increasing the volume of the inner space. The resulting pumping effect could transport microorganisms from the exterior to the interior and vice versa [2, 12–14].

The aim of the present systematic review was to evaluate mechanical outcomes, microleakage, and marginal accuracy at the implant-abutment interface of original versus nonoriginal implant abutments.

## 2. Materials and Methods

The present systematic review was written according to the Preferred Reporting Items for Systematic Reviews and Meta-Analyses (PRISMA) guidelines [15]. The focused question was to identify whether there is a difference at the implant-abutment interface in mechanical outcomes, microleakage, and marginal accuracy between original and nonoriginal implant abutments. To define the search strategy, PICOS questions (Population (P), Intervention (I), Comparison (C), Outcomes and study design (O), and Study type (S)) were

  P: samples of implant-supported single crowns;
  I: original stock or computer-assisted design and computer-assisted manufacturing (CAD/CAM) abutments;
  C: nonoriginal abutments (certified or compatible);
  O: mechanical outcomes (mechanical deformation, damage, and failure under applied forces), microleakage (microgap), and marginal accuracy (fit).
  S: in vitro studies.

Original and nonoriginal abutments were defined as follows:

 
**Original abutment** is an abutment produced by the same implant manufacturer based on original nominal dimensions. These could be stock or CAD/CAM.
 
**Nonoriginal abutment **is an abutment produced by a different implant manufacturer (or third-party company). These could be certified or compatible.
 
**Nonoriginal certified abutment** is an abutment produced by a third-party milling center, directly or indirectly certified for certain implant companies. These could be gold cast or CAD/CAM.
 
**Nonoriginal compatible abutment** is an abutment produced by an implant company compatible with other implant systems with the same implant-abutment interface. It might also be manufactured by a generic producer not directly involved in the implant manufacturing. These could be stock or CAD/CAM.

### 2.1. Search Strategy

An initial search strategy encompassing the English literature from 1967 up to February 2018 was performed online to identify relevant studies that met the inclusion criteria. The following electronic databases were consulted: PubMed database of the U.S. National Library of Medicine and Embase (Excerpta Medica dataBASE). According to the AMSTAR (A MeaSurement Tool to Assess Systematic Reviews) checklist, the Grey Literature Database was screened in the New York Academy of Medicine Grey Literature Report in order to find possible unpublished works. Screening was performed by an expert examiner (MT). A second reviewer (LC) reassessed the included and excluded studies. The electronic databases were searched using a combination of Boolean keywords, MeSH, and several free-text terms. The Boolean search algorithm employed to find potentially relevant literature was developed by extracting the keywords of relevant literature found on preliminary scoping searches and included the following terms: (“Dental Implant-Abutment Design”[Mesh] OR “Dental Implant-Abutment*∗*” OR “Dental Implant Abutment*∗*”) AND (“Computer-Aided Design”[Mesh] OR “*∗*original” OR “*∗*nonoriginal” OR “*∗*nonoriginal” OR “compatible” OR “avatar”).

### 2.2. Eligibly Criteria

The following inclusion criteria were defined for the articles selection:

- written in English;
- comparison between original and nonoriginal abutments;
- in vitro studies;
- systematic reviews, narrative reviews, and consensus statements of in vitro studies.

Articles were excluded if they were animal studies, finite element analysis studies, or editorials.

### 2.3. Data Extraction and Assessment of Quality, Heterogeneity and Risk of Bias

The calibrated reviewers screened and collected the data from selected papers onto structured tables. Articles without abstracts, but with titles related to the objectives of the present review were selected, and full text was screened for eligibility. Additionally, hand searches of the reference lists of selected relevant articles were conducted for inclusion of possible additional papers. The following information was sought and recorded on data extraction forms: name of the author and year of publication, type of implant-abutment interface, number and type of abutments in each group (stock versus CAD-CAM, certified versus compatible) (Table 1).

The following outcome measures were analyzed: (1) mechanical outcomes (mechanical deformation, damage, and failure under applied forces); (2) microleakage (microgap at the implant-abutment interface); (3) marginal accuracy (fit). The same reviewers assessed the quality of the searched manuscript and the risk of bias in the included studies according to a modification of the guidelines provided by the CONSORT statement for the evaluation of randomized controlled trials [16].

## 3. Results

### 3.1. Study Selection

**Figure 1:**
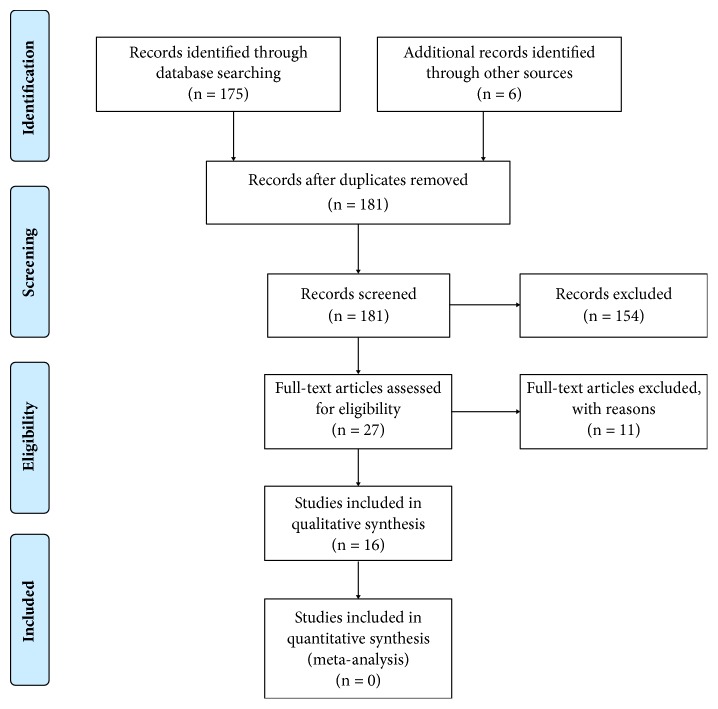
Flow diagram of the search strategy for study selection based on PRISMA.

A total of 175 potentially relevant titles and abstracts were found after the electronic search. During the first stage of selection, 154 articles were excluded based on the titles and abstracts. In the second phase, complete full-text articles of the remaining 21 publications were evaluated and 11 articles were excluded since they did not fulfill the inclusion criteria. Six publications were added from manual extraction of the reference lists of selected relevant articles. Finally, a total of 16 articles that fulfilled inclusion criteria and quality assessment required for the present systematic review, reporting data from 653 implant abutments, were selected in the qualitative analysis. A diagram of the search strategy is shown in Figure 1.

### 3.2. Study Characteristics and Risk of Bias

The selected studies were published between 2003 and 2017. All the included studies were in vitro research reporting data on single-implant-supported restorations with different implant-abutment interface. Overall, 188 abutments were original: ten studies reported data from original stock abutments (n=108) and seven studies reported data from original CAD/CAM abutments (n=80). Conversely, 465 abutments were nonoriginal: seven studies reported data from nonoriginal, compatible, stock abutments (n=237); five studies reported data from nonoriginal, compatible, CAD/CAM abutments (n=73); and six studies reported data from nonoriginal, certified, CAD/CAM abutments (n=155) (Table 1).

According to a modified CONSORT checklist of items for reporting in vitro studies of dental material, two out of 16 studies were at moderate risk of bias [17, 18], while the other 14 studies were at high risk of bias [11, 12, 19–30] (Table 2). Main reason for this risk level was in vitro nature of the studies. In addition, none of the publications reported sample size calculations. Only three studies used a random allocation sequence [17–19], but none of these reported any information on the mechanism used to implement the random allocation sequence. Moreover, none of the included studies reported any information on the method used to blind the outcome assessors regarding the treatment group assignment. Six studies performed the planned tests according to the ISO 14801:2007 standards for “Dynamic loading test for endosseous dental implants” [17, 18, 22, 24, 27, 28]. Lastly, a majority of the studies utilized titanium abutments [11, 12, 17, 19, 20, 22, 25, 27, 30]; two studies used zirconia abutments [18, 21]; and only one study used both titanium and zirconia abutments [26].

## 4. Outcomes

### 4.1. Mechanical Outcomes

Mechanical outcomes has been defined as mechanical deformation, damage, and failure under applied forces. Gigandet et al. [22] demonstrated that the rotational misfit of a nonoriginal compatible CAD/CAM abutment was higher compared to the original abutments of Straumann implants system. Moreover, they revealed that the combination of grooves and surfaces was completely different between original and nonoriginal abutments [22]. In addition, Berberi et al. [12] observed that, under simulated clinical loading conditions, the use of compatible stock abutments leads to significant micro-movement compared to the use of original abutments.

Four studies [17, 24, 27, 28] evaluated the post-fatigue reverse-torque values at the IAI according to the ISO 14801:2017 standards. Overall, the effect of component manufacturer resulted in a significantly lower reverse-torque value in the nonoriginal compatible abutments indicating greater residual preload. However, there was no significant decrease in post-fatigue reverse-torque value for either original or nonoriginal abutments compared to baseline [17]. Park et al. [28] and Kim et al. [24] demonstrated that under dynamic loading cycles the removal torque of original abutments was significantly higher compared to nonoriginal compatible stock abutments and nonoriginal certified CAD-CAM abutments, respectively. Nevertheless, with precise control of nonoriginal certified CAD/CAM abutments, proper screw joint stability can be achieved [27].

When testing the maximum load capacity, Kim et al. [18] reported different fracture behavior in all of the tested abutments, with significantly higher load capacity for a nonoriginal certified CAD/CAM abutments (Lava Zirconia abutment) produced by third-party company.

Leuter et al. [26] demonstrated that both original and nonoriginal certified CAD/CAM abutments affected the bending moments of abutments after static loading. They found that internally connected zirconia abutments with horizontal mismatch to the implant exhibited significantly higher bending moments compared to those titanium implant-abutment connections, independently by the manufacturers. In another study, no significant differences were found between original stock abutments (OS) and implants connected to nonoriginal laser-sintered abutments in the mechanical behavior under static and dynamic loading conditions [20].

On the contrary, Yilmaz et al. [29], conducting a load to failure comparison of 5 different titanium abutments, observed that the manufacturer's abutments were the only one not involved in any of the components fracture. The authors suggested that screw fractures experienced by the aftermarket brands may result in further clinical prosthetic complications [29].

### 4.2. Marginal Accuracy (Leakage, Fit, Microgap)

Microleakage may be defined as the clinically undetectable passage of bacteria, fluids, molecules, or ions between a cavity wall and the restorative materials. Microleakage directly depends on the marginal accuracy of the components (fit, tolerances, presence of microgaps). Alonso-Pérez et al. [20] evaluated the marginal vertical gap and mechanical outcomes of original and nonoriginal certified CAD-CAM abutments. They concluded that both abutments can be successfully used to restore implants, although the original abutments demonstrated better fit than nonoriginal ones [20]. Hamilton et al. [23] concluded that the original and nonoriginal compatible CAD/CAM abutments appeared to have a comparable fit with original stock abutments for most of the systems evaluated. Some design differences between original and nonoriginal abutments were observed for Straumann implants that affected the fit of internal components of the implant-abutment connections. Solá-Ruiz et al. [30] also reported the compatibility and possibility of combining the different brands of implants and their respective (nonoriginal) compatible stock abutments between different brands of implants with external hexagon and their machined titanium prosthetic abutments. In addition, Lang et al. [25] showed that nonoriginal compatible CAD/CAM Procera abutments can be universally applied to the implant systems studied.

Conversely, in a publication on the analysis of the vertical marginal fit after static and dynamic load, after thermocycling with artificial saliva, the authors found that original abutments were highly superior to nonoriginal certified abutments [19]. Da Cunha et al. [21] observed that the degree of misfit of original abutments was approximately half that observed with nonoriginal compatible CAD-CAM abutments. Nevertheless, the range of misfit reported in the current study is considered by many authors as clinically acceptable. Berberi et al. [11] showed that the use of nonoriginal compatible stock abutments leads to significant higher width gap and micromovements when compared with the use of original ones, potentially improving microbial leakage at the IAI.

### 4.3. Radiographic Marginal Bone Loss and Biological or Mechanical Complications

None of the included studies reported data from radiographic marginal bone loss, as well as biological or mechanical complications due to the nature of the selected study design.

## 5. Discussion

The aim of the present systematic review of in vitro studies was to evaluate any difference in mechanical outcomes, microleakage, and marginal accuracy between original and nonoriginal abutments in implant-supported restorations. In fact, dental implant abutments can be either original or nonoriginal. Original abutments are manufactured by the same implant company. Recently, due to the wide range of customized abutments request, international milling centers proposed “certified” nonoriginal connections. At the same time, original designs have been incorporated in a various number of competing companies that offer the so-called “compatible” connections, and their relative nonoriginal, compatible, dental implant abutments. None of the included studies reported data from radiographic marginal bone loss, as well as biological or mechanical complications.

To the best of our knowledge, at the time of writing this systematic review, there were no in vitro studies comparing mechanical outcomes and marginal accuracy at the implant-abutment interface of original versus nonoriginal implant abutments. Furthermore, with so many variables of implant types, connections, materials, methods of manufacture of the restorations existing, it is not possible to combine data from multiple studies.

The majority of the included studies [11, 17, 22, 28] reported lower values of mechanical resistance for nonoriginal compatible stock abutments. Nevertheless, controversial outcomes were reported by Yilmaz et al., which supported the possibility of combining nonoriginal compatible stock abutments between different brands of implants. It must be highlighted, however, that these outcomes were reached using the easiest connection available on the market (external hexagon), which of course level off mechanical outcomes, failing to show differences [29]. On the contrary, nonoriginal certified abutments showed results on mechanical outcomes similar to original abutments [18, 20, 26, 27].

Although reported exponential source of bias (heterogeneity across the included trials, together with confounding factors) makes the translation to the clinical practice difficult, clinical relevance of this review could be extrapolated. In fact, dimensional tolerances express the possible space between two connecting components and the permissible limits of variation in a physical dimension deviating from a nominal dimension as noted in this review. The tolerances between implant and abutment may play a major role in an implant-abutment connection, defining its mechanical outcomes. Although high quality manufacturing cannot prevent tolerances, data collected in the present systematic review might suggest that differences between original and nonoriginal certified or noncertified abutments might be defined by the size of implant/abutment tolerances.

In two-piece implant, a microgap is created at the implant-abutment interface. Previous systematic review and meta-analysis have shown that oral microbiome can proliferate in this microgap and affect peri-implant tissues, causing inflammation and peri-implant diseases [10]. Preventing microbial leakages through is therefore an important goal in implantology. From a clinical perspective, the size of the microgap at the implant-abutment interface may contribute to bone remodeling after implant placement [6, 31]. Bacterial colonization of the gap at the implant-abutment interface has been implicated in this process, influencing the supracrestal soft tissue attachment (previously called “biologic width establishment”) [32]. Additionally, it has been suggested that the microleakage at the gap between the implant and the abutment may represent a path for acids, enzymes, bacteria, and/or their metabolic products that directly affect the periodontal tissue, triggering the inflammatory response [4]. High quality manufacture and quality controls produce components with precise fit and ideal load distribution. This prerogative might help to ensure maintenance of crestal bone and long-lasting esthetics.

The results of this review also demonstrate that CAD/CAM abutments provide comparable, if not better, clinical outcomes when compared, in vitro, with conventional abutments. However, existing evidence is weak. Schepke et al., in an in vivo randomized controlled trial comparing the use of a CAD/CAM customized zirconia abutments with stock zirconia abutments, for the rehabilitation of a single tooth replacement, failed to find any improvement in clinical performance or patients' satisfaction [33].

The main limitation of the present systematic review was the presence of only in vitro studies reporting insufficient methodological details. Therefore, due to the in vitro nature of these studies and the high risk of bias, the outcomes reported in these systematic reviews should be carefully interpreted. In fact, the in vitro values do not represent the clinical situation because only vertical load application can be used for the comparative analysis, and the numerous biological parameters that influence mechanical outcomes in vivo are not taken into account [34, 35]. Long-term randomized controlled trials with two types of abutment (original versus nonoriginal) will be needed to help decision making.

Additional limitation might be represented by the fact that none of the included studies reported data from multiple implants, not allowing a complete generalization of the study outcomes.

In the light of this preclinical outcomes, long-term consequences of nonoriginal abutments should be analyzed to evaluate the clinical relevance of their mechanical instability. For this purpose, long-term randomized controlled trials aimed to compare original to nonoriginal restorations and the microbiological contamination at the connection level could answer this clinical question.

## 6. Conclusions

Lower incidence of mechanical failure and higher marginal accuracy were reported for originals compared to nonoriginal abutments. Differences between nonoriginal certified and nonoriginal compatible were highlighted. However, clinical conclusions regarding the effect of a nonoriginal abutment cannot be drawn. In fact, data were derived from “in vitro” studies with a high risk of bias.

Therefore, there is an urgent need for well-designed randomized clinical trials to provide more information about the long-term clinical outcomes of original and nonoriginal abutments.

## Figures and Tables

**Table 1:** Main data of the included studies.

Authors	IAI	Abutments Test + Control	Test Original Abutments	Control Non-original abutments
Alonso-Pérez et al. 2017a	IH	45	Stock (n=15)	Compatible stock (n=30)

Alonso-Pérez et al. 2017b	IH	26	Stock (n=13)	Certified CAD/CAM (n=13)

da Cunha et al. 2012	MT	24	CAD/CAM (n=8)	Compatible CAD/CAM (n=16)

Berberi et al. 2014	MT	28	Stock (n=7)	Compatible stock (n=21)

Berberi et al. 2016	MT	15	Stock (n=5)	Compatible stock (n=10)

Cashman et al. 2011	EH	40	Stock (n=20)	Compatible Stock (n=20)

Gigandet et al. 2014	MT,I3L	60	CAD/CAM (n=36)	Compatible CAD/CAM (n=24)

Hamilton et al. 2013	MT,EH,I3L	11	Stock (n=6) CAD/CAM (n=1)	Compatible CAD/CAM (n=4)

Kim et al. 2013	I3L	60	CAD/CAM (n=20)	Certified CAD/CAM (n=40)

Kim et al. 2013	MT	21	Stock (n=7)	Certified CAD/CAM (n=7) Certified gold cast (n=7)

Lang et al. 2003	EH	30	CAD/CAM (n=6)	Compatible CAD/CAM (n=24)

Leutert at al. 2012	MT	84	CAD/CAM (n=4)	Certified CAD/CAM (n=80)

Paek et al. 2016	MT	6	Stock (n=3)	Certified CAD/CAM (n=3)

Park et al. 2017	MT	28	Stock (n=7)	Compatible stock (n=21)

Sola-ruitz et al. 2013	EH	150	Stock (n=25)	Compatible stock (n=125)

Yilmaz et al. 2015	IH	25	CAD/CAM (n=5)	Compatible stock (n=10) Compatible CAD/CAM (n=5) Certified CAD/CAM (n=5)

Total		653	188 (Stock 108, CAD/CAM 80)	465 (Certified 155, Compatible 310)

**Legend**: IH: internal hexagon; MT: morse tapered; EH: external hexagon; I3L: internal tri-channel; IAI: implant-abutment interface.

**Table 2:** Reporting quality of all included studies.

Authors	Trial design	How sample size was determined	Random allocation sequence	Mechanism used to implement the random allocation sequence	Method for blinding outcome assessors	ISO 14801	Risk of bias
Alonso-Pérez et al. 2017a	YES	NR	YES	NR	NR	NR	High

Alonso-Pérez et al. 2017b	YES	NR	NR	NR	NR	NR	High

da Cunha et al. 2012	YES	NR	NR	NR	NR	NR	High

Berberi et al. 2014	YES	NR	NR	NR	NR	NR	High

Berberi et al. 2016	YES	NR	NR	NR	NR	NR	High

Cashman et al. 2011	YES	NR	YES	NR	NR	YES	Moderate

Gigandet et al. 2014	YES	NR	NR	NR	NR	YES	High

Hamilton et al. 2013	YES	NR	NR	NR	NR	NR	High

Kim et al. 2013	YES	NR	YES	NR	NR	YES	Moderate

Kim et al. 2013	YES	NR	NR	NR	NR	YES	High

Lang et al. 2003	YES	NR	NR	NR	NR	NR	High

Leutert at al. 2012	YES	NR	NR	NR	NR	NR	High

Paek et al. 2016	YES	NR	NR	NR	NR	YES	High

Park et al. 2017	YES	NR	NR	NR	NR	YES	High

Sola-ruitz et al. 2013	YES	NR	NR	NR	NR	NR	High

Yilmaz et al. 2015	YES	NR	NR	NR	NR	NR	High
